# Life negative events and depressive symptoms: the China longitudinal ageing social survey

**DOI:** 10.1186/s12889-020-09119-0

**Published:** 2020-06-19

**Authors:** Zhenjie Wang, Hanmo Yang, Pianpian Zheng, Bei Liu, Zhanyuan Guo, Shen Geng, Shenda Hong

**Affiliations:** 1 Institute of Population Research, Peking University, No. 5 Yiheyuan Road, Haidian District, Beijing, 100871 People’s Republic of China; 2 National School of Development, Peking University, No. 5 Yiheyuan Road, Haidian District, Beijing, 100871 People’s Republic of China; 3 School of Computational Science and Engineering, Georgia Institute of Technology, 756 W Peachtree St NW, Atlanta, 30308 Georgia USA

**Keywords:** Older adults, Depressive symptoms, Life negative events exposure, China

## Abstract

**Background:**

Although some studies have reported the association between life negative events and depressive disorders, very limited studies have examined the association between life negative events exposure and depressive symptoms risk among Chinese older adults.

**Methods:**

Data were obtained from the China Longitudinal Ageing Social Survey (CLASS), which was a stratified, multi-stage, probabilistic sampling survey, conducted in 2014. General linear regression and logistic regression were used to examine the association between life negative events exposure and depressive symptoms among Chinese older adults.

**Results:**

Life negative events showed statistical dose-response association with depressive symptoms risk after adjustment for the confounding factors (*P*_trend_ < 0.001). Under consideration of life negative events exposure, participants who lived in rural areas, without a spouse or live alone were vulnerable to depressive symptoms.

**Conclusions:**

Life negative events played a risk role of depressive symptoms among Chinese older adults, especially among those in rural areas, females or without a spouse. Our current study is valuable for the development of special prevention depressive symptoms programs among elderly individuals, especially those who have experienced negative events.

## Background

Depressive symptoms (DS), which is characterized by symptoms of sadness, depressed mood, and loss of interest, is one of the most prevalent mental disorders across the world [1]. People with DS are usually associated with low quality of life, prevalence of cancer, chronic diseases, suicide, and mortality [2–5]. DS could place a heavy burden on families, communities and health services in both high-income countries and low- and middle-income countries [6, 7].

The prevalence of DS varies widely across countries, from 1 to 16% among middle-aged and elderly people in studies conducted in developed countries [8–10]. As China is facing rapid urbanization and many environmental challenges, the prevalence of DS has been increasing rapidly from 3.9 to 17% among elderly Chinese people in past two decades [11–13].

Several socioeconomic variables were presented to be associated with DS risk [14], although the associations are inconsistent [10, 13]. A number of studies found inverse or U-shaped associations between age and DS [15–17]. Moreover, low socioeconomic status was found to be associated with severe DS in both men and women, and low education level was found to be related with high risk of DS [18–20]. However, some studies reported no association between income and DS [21] or between education level and DS [14, 17].

Studies have proved that exposure to life negative events, such as serious illnesses, injuries, deaths of loved ones, marital separations, break-ups of steady relationships, onset of unemployment, major financial crises, and so on, is associated with depressive disorders and anxiety [22, 23]. However, there is limited epidemiologic evidence linking life negative events exposure with DS risk among Chinese older population. Therefore, we investigated the association of recent negative events exposure, including serious diseases (self or family member), natural disaster/accident, the death of a family member (spouse, children, or relatives), with depressive symptoms risk using data from the Chinese Longitudinal Ageing Social Survey (CLASS).

## Methods

### Setting and sample

This survey used a stratified, multi-stage, probabilistic sampling method to select nationally representative sample. Details of the study design and conduct were described elsewhere [24]. There were 11,511 older adults were surveyed in total. In the present study, the sample comprised 8711 subjects aged 60 years or older who answered the questions on depressive symptoms and other independent variables of interests, such as demographic variables, health, health serveries, socioeconomic variables, social support and so on. All the participants were interviewed face-to-face by trained interviewers. During the interview, interviewers should choose an independent and quiet environment, avoid the presence of irrelevant people.

### Measurement

Depressive symptoms were assessed by using a nine-item Center for Epidemiological Studies Depression Scale (CES-D), including three items assessed positive feelings, two items assessed negative emotions, two items assessed somatic symptoms, and two items assessed sense of marginalization. 9-item CES-D was reliable and valid for detecting non-psychotic mental disorders among Chinese older adults [25]. Each item had a score of 0 (rarely or none of the time), 1 (some of the time), or 2 (most of the time), with the total score ranging from 0 to 18. By reversing the coding of the positive effect items, a higher score indicates a higher level of depressive symptoms. For the current study, on a 9-item scale, the total possible score is 18 (9 items multiplied by 2, the highest response). That total score is divided by 60 (the total possible score on the full 20-item CES-D), which equals 0.3 [26]. Then, the 0.3 is multiplied by 16, resulting in a standardized cut score of 4.8 for the 9-item form of the CES-D. In this study, the internal Cronbach’s alpha for the nine items was 0.75.

In the current study, the life negative events exposure was the experience of a life negative event reported by the subjects themselves in the past 12 months. The information of the seven life negative events, including serious diseases (self or family member), natural disaster/accident, the death of a family member (spouse, children, or relatives), were collected during the survey. Number of life negative events in past 12 months were counted and further categorized (“0” = 0, “1” = 1, “≥2” = 2). Socio-demographic characteristics included gender (male, female), age, marital status (married, widowed/divorced/unmarried), ethnicity (Han, others), residence (rural, urban), education level (junior high school and above, primary school, never attended school), and living arrangements (lives alone, lives with others). Ten-item version of the activities of daily living (ADL) was assessed for physical disability [27]. Chronic diseases, including any health problems: hypertension, diabetes, heart disease, renal disease, liver disease, stroke, tuberculosis, arthritis, respiratory and so on, were categorized into “yes” = 1, and “no” = 0.

### Statistical analysis

Mean ± SD (standard deviation) was used for the description of continuous variables and percentage for categorical data. General linear regression was used to examine the association between depressive symptom score and number of life negative events in the past 12 months. Logistic regression analysis was used to estimate odds ratios (OR) and 95% confidence intervals (CI) of depression risk for each category with the lowest category as the reference group. Trends of the associations were assessed with ordinal scores assigned to categories of the number of life negative events in the past 12 months. Another analysis was done according to socio-demographical status, gender (male, female), residence (rural, urban) and living arrangements (lives alone, lives with others) to examine associations between the number of negative events in the past 12 months and depression risk under considering confounding variables. Statistical significance was declared with a two-sided *p*-value < 0.05. Statistical analyses were performed using SAS version 9.4 (SAS Institute Inc., Cary, NC, USA).

## Results

Selected characteristics of subjects with/without depressive symptoms (DS) are summarized in Table 1. The overall average Center for Epidemiological Studies Depression Scale (CES-D) score was 4.56 (Standard Deviation: 3.56). The prevalence of DS was 43% among Chinese population aged over 60 years. In the current study, male subjects, urban residents, people living with others, and Han nationality accounted for the majority proportion among Chinese old population.

**Table 1:** Characteristics distribution among elder population in China

Variables	Sample size (*n* = 8177)	Depressive symptoms	
		with depressive symptoms (*n* = 3542)	without depressive symptoms (*n* = 4635)
Age (years), mean (sd)	69.07 (7.47)	69.53 (7.67)	68.73 (7.33)
Education level			
Junior High school and above	1670	931 (26.29%)	739 (15.94%)
Primary school	2948	1408 (39.75%)	1540 (33.23%)
Never attended school	3559	1203 (33.96%)	2356 (50.83%)
Gender (%)			
Male	4378	1800 (50.82%)	2578 (55.62%)
Female	3799	1742 (49.18%)	2057 (44.38%)
Marital status (%)			
Married	5810	2258 (63.75%)	3552 (76.63%)
Widowed/divorced/unmarried	2367	1284 (36.25%)	1083 (23.37%)
Ethnicity			
Han	7659	3291 (92.91%)	4368 (94.24%)
Others	518	251 (7.09%)	267 (5.76%)
Residence			
Rural	2801	1487 (41.98%)	1314 (28.35%)
Urban	5376	2055 (58.02%)	3321 (71.65%)
Living arrangement (%)			
Live with others	7200	2972 (83.91%)	4228 (91.22%)
Live alone	977	570 (16.09%)	407 (8.78%)
Physical disability			
No function problems	7080	2799 (79.02%)	4281 (92.36%)
One and more functioning limitations	1097	743 (20.98%)	354 (7.64%)
Chronic diseases (%)			
Yes	5933	2909 (82.13%)	3024 (65.24%)
No	2244	633 (17.87%)	1611 (34.76%)
Number of life negative events in past 12 months			
0	5945	2328 (65.73%)	3617 (78.04%)
1	1792	925 (26.11%)	867 (18.70%)
≥ 2	440	289 (8.16%)	151 (3.26%)

The associations of the number of negative events with DS risk in Table 2. The number of negative events showed statistical association with depression risk after adjustment for the confounding factors (*P*_trend_ < 0.001). Residual confounding by residence area, gender, and living arrangement might be potential concerns because these are positive risk factors for depression. We further analyzed the association of the number of negative events (Table 3) with depression stratified by selected covariates (i.e., residence area, gender, living arrangement, and physical disability). Increased risk of DS was noted in selected covariates. We found the elders who were living in urban areas decreased 30% risk of DS compared with those lived in rural areas among the elders without any types of negative event. Among the highest category of number of life negative events in past 12 months, the elders who lived in urban areas was lower risk than those who lived in rural areas. The elders who lived alone increased 56% risk of DS than those who lived with others among the elders without any types of negative event. Old population without a spouse presented a higher risk of DS than those live with a spouse, and the difference became larger as they experienced more life negative events.

**Table 2:** Odds ratio (95% confidence interval) of depressive symptoms risk according to number of life negative events

	Number of life negative events in past 12 months			*P*_trend_
	0	1	≥2	
With DS/ Without DS	2328/3617	925/867	289/151	
DS score, mean (sd)	4.19 (3.36)	5.27 (3.74)	6.62 (4.16)	< 0.001
OR (95% CI)^a^	1 (reference)	1.54 (1.38–1.71)	2.57 (2.08–3.16)	< 0.001
OR (95% CI)^b^	1 (reference)	1.35 (1.21–1.52)	2.05 (1.65–2.54)	< 0.001

*Abbreviations*: *DS* depressive symptoms, *CI* confidence interval, *OR* odds ratio

^a^ Adjusted for age, gender, residence area, education level, marital status, ethnicity and living arrangement

^b^ Further adjusted for physical disability and chronic diseases

**Table 3:** Odds ratio (95% confidence interval) of depressive symptoms risk according to number of life negative events stratified by selected covariates^a^

	Number of life negative events in past 12 months			*P*_trend_	*P*_interaction_
	0	1	≥2		
Residence area ^b^					
Rural	1.00 (reference)	1.22 (1.02–1.46)	1.97 (1.46–2.66)	< 0.001	0.33
Urban	0.70 (0.62–0.79)	1.01 (0.86–1.19)	1.46 (1.06–2.01)	< 0.001	
Gender ^c^					
Male	1.00 (reference)	1.32 (1.14–1.54)	1.65 (1.25–2.17)	< 0.001	0.06
Female	0.96 (0.85–1.07)	1.33 (1.12–1.57)	2.71 (1.91–3.85)	< 0.001	
Marital status ^d^					
Married	1.00 (reference)	1.42 (1.24–1.62)	1.91 (1.50–2.44)	< 0.001	0.93
Widowed/divorced/unmarried	1.46 (1.27–1.67)	1.77 (1.45–2.17)	3.84 (2.42–6.11)	< 0.001	
Living arrangement ^e^					
Live with others	1.00 (reference)	1.35 (1.19–1.52)	2.06 (1.64–2.59)	< 0.001	0.96
Live alone	1.56 (1.30–1.88)	2.21 (1.64–2.98)	3.01 (1.62–5.59)	0.008	

*Abbreviations*: *CI* confidence interval, *OR* odds ratio

^a^ Adjusted for age, gender, residence area, education level, marital status, ethnicity, living arrangement, physical disability and chronic diseases

^b^ Models did not include residence area

^c^ Models did not include gender

^d^ Models did not include marital status

^e^ Models did not include living arrangement

## Discussion

Overall, the prevalence of depressive symptoms (DS) was 43% among Chinese old population. The result shows that the risk of depression is related to the number of life negative events experienced among Chinese older population.

In China, a large number of studies concerning DS in older adults have emerged [12, 28]. However, the results of prevalence of DS among older adults varied from 6.4% [29] to 60.3% [30], which is due to the inconsistent criteria of the measuring tools used and differences in sample sizes and sociocultural contexts. Our prevalence was similar to an observational study [31]. A previous meta-analysis had estimated the prevalence of depressive symptoms (23.6%) in Chinese older adult population [12]. It suggested that the prevalence of DS in Chinese older adults has been increasing significantly [12].

Our finding supports the knowledge that adults exposed to at least one negative event might experience trauma-related mental health conditions [32]. Previous studies suggested that the strength and scale of associations between demographic factors and depression among older adults were different across genders [33–35]. Our results also indicate that female gender is positively associated with having depressive symptoms in such that elderly female adults were 1.6 times (2.71/1.65) more likely to have depressive symptoms than males who have experienced two or more life negative events in the past 12 years, which was in line with the other findings in developing countries [33, 36–38]. The reasons why females were more affected by depression than males could be attributed to biological or environmental factors [12, 39, 40].

Marital status and living arrangement were regarded as very influential factors for depression among Chinese older adults with the number of negative events experienced under consideration. Widowed, divorced or separated older adults have experienced more stressful life events and have a relatively higher level of loneliness, which is one of the manifestations of depression [12, 41, 42]. Moreover, family member’s support was a significant indicator of DS of older adults [12]. A spouse or other family members can take care of and talk to his or her partner who is experiencing stressful/ negative events as much as he or she can receive emotional and spiritual support from his or her partner, which may reduce depression [12].

The strength of current study is that the CLASS was a population-based survey with a large sample. However, our study also has several limitations, which should be a consideration for further researchers. Firstly, the cross-sectional design of the baseline of the CLASS does not provide direct evidence of causality. We will be able to extend the current study to determine causality when longitudinal data are available. Moreover, due to data limitations, only the past 12 months’ negative events and types of negative events were collected in this study. We could not assess the frequency, length, or intensity of negative events during lifetime, which should be cautioned in future studies. The CLASS was also lack of information on other mental disorders except for depressive symptoms, which should be cautioned in future studies. Additionally, depressive symptoms were different from diagnosis of depressive disorder, that should be noticed for researchers. Lastly, the CLASS does not provide sufficient information on weight, height, smoking, alcohol drinking, and so on, which were suggested as risk factors of depression.

## Conclusions

In conclusion, in this large population-based study among Chinese older population, we found that 43% of the elders are experiencing depression. Family-related negative events exposure were associated with depression risk and presented dose-response associations. Moreover, characteristics presented the marginally modified association with depression in the current study. Findings of our study are valuable for the development of prevention programs in identifying elderly individuals, who were exposed to life negative events.

## Data Availability

The datasets generated and/or analysed during the current study are available in the China Longitudinal Aging Social Survey (CLASS) repository, http://class.ruc.edu.cn/index.php?r=index/index&hl=en.

## Abbreviations

CLASS: The China Longitudinal Ageing Social Survey
CES-D: Center for Epidemiological Studies Depression Scale
